# Supplementary material for: Cenobamate (YKP3089) and Drug-Resistant Epilepsy: A Review of the Literature
Source: Medicina (Kaunas). 2023 Jul 28;59(8):1389. doi: 10.3390/medicina59081389 (PMC10456719; doi:10.3390/medicina59081389)
Supplement: Supplementary file 1 [file medicina-59-01389-s001.zip › medicina-2451890-supplementary.pdf]

medicina

MDPI

# Supplementary Matherial: Cenobamate (YKP3089) and Drug-Resistant Epilepsy: A Review of the Literature

**Jamir Pitton Rissardo and Ana Letícia Fornari Caprara**

## Methodology

Search Strategy

We searched the PubMed database to locate the studies on cenobamate published in May 2023 in electronic form. Search terms were "cenobamate, xcopri, ontorzy, and YKP3089." No language restriction was applied. There was one publication in German, all the other articles were in English.

**Table S1.** PubMed database search.

| | | |
|---|---|---|
| (cenobamate) OR (xcopri) OR (ontorzy) OR (YKP3089) | "cenobamate"[Supplementary Concept] OR "cenobamate"[All Fields] OR "cenobamate"[All Fields] OR "cenobamate"[Supplementary Concept] OR "cenobamate"[All Fields] OR "cenobamate"[All Fields] OR "xcopri"[All Fields] OR "cenobamate"[Supplementary Concept] OR "cenobamate"[All Fields] OR "ykp3089"[All Fields] | 110 results |

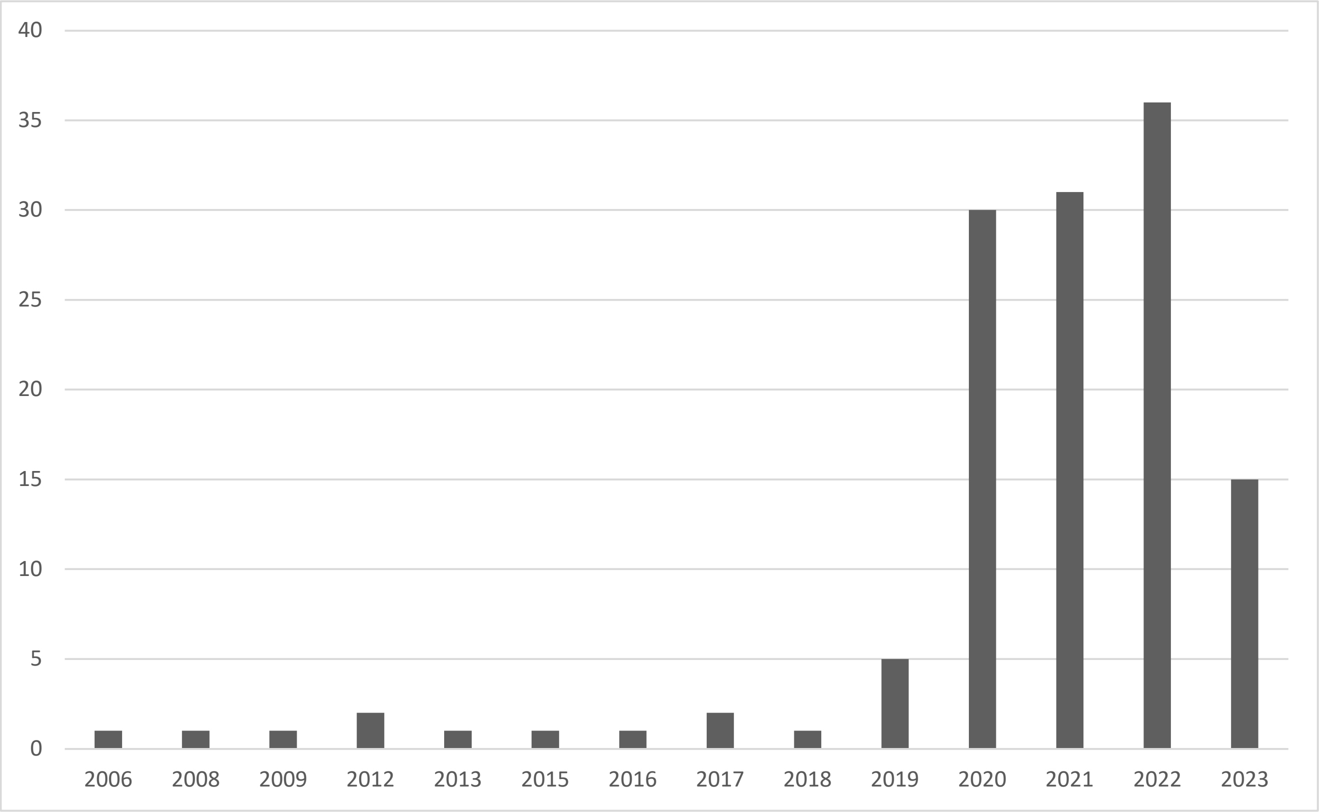


**Figure S1.** Number of publications in PubMed over time.
